# The Inactivation by Curcumin-Mediated Photosensitization of *Botrytis cinerea* Spores Isolated from Strawberry Fruits

**DOI:** 10.3390/toxins13030196

**Published:** 2021-03-09

**Authors:** Li Huang, Ken W. L. Yong, W. Chrishanthi Fernando, Matheus Carpinelli de Jesus, James J. De Voss, Yasmina Sultanbawa, Mary T. Fletcher

**Affiliations:** 1 Institute of Light Industry and Food Engineering, Guangxi University, Nanning 530004, China; mary607@126.com; 2 Queensland Department of Agriculture and Fisheries, 39 Kessels Rd, Coopers Plains, QLD 4108, Australia; ken.yong@daf.qld.gov.au; 3 Queensland Alliance for Agriculture and Food Innovation (QAAFI), The University of Queensland, 39 Kessels Rd, Coopers Plains, QLD 4108, Australia; chrishanthif2@gmail.com; 4 School of Chemistry and Molecular Biosciences, The University of Queensland, St Lucia, QLD 4072, Australia; m.carpinellidejesus@uq.edu.au (M.C.d.J.); j.devoss@uq.edu.au (J.J.D.V.)

**Keywords:** photodynamic therapy, botryane, secondary metabolites, dihydrobotrydial, fungal spoilage

## Abstract

Photosensitization is a novel environmentally friendly technology with promising applications in the food industry to extend food shelf life. In this study, the natural food dye curcumin, when combined with visible light (430 nm), was shown to be an effective photosensitizer against the common phytopathogenic fungi *Botrytis cinerea* (the cause of grey mould). Production of the associated phytotoxic metabolites botrydial and dihydrobotrydial was measured by our newly developed and validated HRAM UPLC-MS/MS method, and was also shown to be reduced by this treatment. With a light dose of 120 J/cm^2^, the reduction in spore viability was directly proportional to curcumin concentrations, and the overall concentration of both botrydial and dihydrobotrydial also decreased with increasing curcumin concentration above 200 µM. With curcumin concentrations above 600 µM, the percentage reduction in fungal spores was close to 100%. When the dye concentration was increased to 800 µM, the spores were completely inactive and neither botrydial nor dihydrobotrydial could be detected. These results suggest that curcumin-mediated photosensitization is a potentially effective method to control *B. cinerea* spoilage, and also to reduce the formation of these phytotoxic botryane secondary metabolites.

## 1. Introduction

Strawberries are one of the most popular fruits consumed worldwide, with global production in excess of 9M tons per annum and an estimated annual production value of USD 18B worldwide [1]. These fruits are rich sources of antioxidants and vitamin C [2], but their commercial value is restricted by limited shelf-life due largely to susceptibility to microbial infections, particularly *Botrytis* grey mould. *Botrytis cinerea* is the causal agent of this disease, which effects not only strawberries, and is considered one of the most important phytopathogens of horticultural crops. This fungus is found in more than 200 ornamental and agriculturally important plants in the world, and ranks second in the list of ‘top 10’ fungal plant pathogens [3].

Antimicrobial photosensitization is an effective and promising approach to elicit cell death and kill microorganisms such as Gram-negative and Gram-positive bacteria and yeasts, as well as parasites and viruses [4,5,6]. The basic principle of photosensitization is a photochemical process in which a photosensitizer dye such as crystal violet, methylene blue and safranin O are energized to an unstable singlet excited-state after absorbing light photons, and then loses its excess energy to produce free radicals including superoxide and hydroxyl radicals (Type I pathway) and singlet oxygen (Type II pathway). These reactive oxygen species (ROS) cause oxidative damage to biomolecules including amino acids, lipids, nucleic acids, with ensuing cell death [7,8]. The application of natural plant additives such as curcumin as photosensitizer is considered a clean and green technology with minimal reported adverse impacts [9]. By comparison fungicides, and other methods, such as irradiation, biocontrol agents, etc., that are currently used to extend food shelf life, show unfavourable side effects that can impact on consumer health. Photosensitization is thus being investigated as a novel technology to accomplish microbiological decontamination and extend the shelf life of food in an environmentally friendly way.

**Figure 1:**
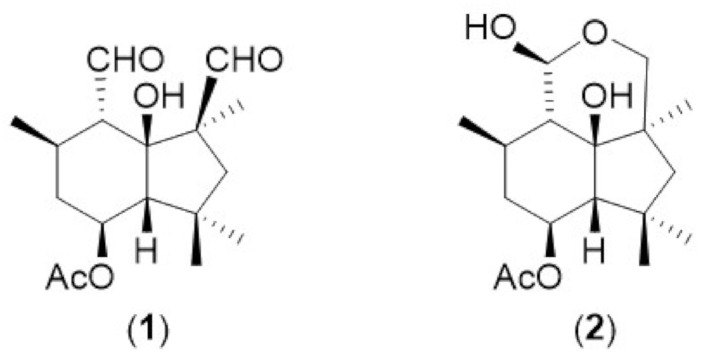
Chemical structures of botrydial (**1**) and dihydrobotrydial (**2**).

*B. cinerea* has many unique characteristics in its phytopathological behaviour, including the capacity to kill host cells through both the production of toxins and also oxidative burst (reactive oxygen species) generated by both the host and the pathogen [10]. This fungus is both a pathogenic and saprophytic organism, initially infecting the weak or dead parts of the plant, and then extending to the rest of healthy plant tissue [11]. *B. cinerea* produces characteristic fungal metabolites based on the botryane skeleton, principally botrydial (**1**) and its metabolite dihydrobotrydial (**2**) (Figure 1), which have been isolated from fungal culture medium [12]. Botrydial is a particularly powerful phytotoxin, produced during plant infection, which can induce chlorosis and cell collapse. It has been demonstrated to have phytotoxic and cytotoxic activity at ID_50_ as equal to or lower than 5 µg/mL and antibiotic activity at 100 ppm [13]. A further *B. cinerea* phytotoxin botcinic acid has also been studied and shown to have a redundant role in virulence with botrydial [14].

Even though photosensitization has been demonstrated to have significant anti-microbial efficiency against an array of microorganisms, there is limited literature available as to whether photosensitization will inhibit the growth of *B. cinerea*, and moreover the impacts of photosensitization on the production of the phytotoxic metabolites botrydial and dihydrobotrydial. Previous studies have investigated the antifungal effect of 405 nm light at 60 mW cm^−2^ for 24 h on this phytopathogen [15]. These authors suggested that the excitation of endogenous porphyrins and subsequent accumulation of singlet oxygen contribute to the light-mediated photoinactivation of grey mould, and suggested such treatment as a means of controlling plant disease. Such lengthy irradiation would, however, not be appropriate with a food commodity such as strawberries, and moreover these authors did not examine the impacts of this treatment on the phytotoxic metabolites.

In this study, *B. cinerea* was isolated from Queensland (Australia) grown strawberries and used to evaluate the effects of photosensitization on the growth of this fungus under a range of experimental conditions including different concentrations of photosensitizer (curcumin), incubation time, and light dose with irradiation periods of 10–30 min. At the same time, a new high-resolution accurate mass (HRAM) UPLC-MS/MS method was developed and validated to study the relative formation of the metabolites botrydial and dihydrobotrydial under these photosensitization conditions.

## 2. Results

### 2.1. Extraction of Botrydial and Dihydrobotrydial from B. cinerea Cultures

Solvent extraction of *B. cinerea* cultures obtained from Queensland strawberries provided a mixed sample of the fungal metabolites botrydial and dihydrobotrydial after repeated silica chromatography. Unfortunately, attempts to further purify these compounds resulted in further degradation of these metabolites. Botrydial and dihydrobotrydial are known compounds for which both ^1^H and ^13^C NMR have been reported [16,17], and literature data were used to both confirm the identity of the two components in our extract and also enabled quantification of the amount of each component within the mixed sample. Integration of ^1^H NMR resonances with a measured aliquot of dioxane as internal standard were used to determine the amount of botrydial/dihydobotrydial present in the sample. The isolated mixed standard was determined to contain botrydial and dihydrobotrydial in a 1:9 ratio, and used without further purification as a mixed external standard in the development of and validation of a new HRAM UPLC-MS/MS analysis method for both metabolites.

### 2.2. HRAM UPLC-MS/MS Analysis of Botrydial and Dihydrobotrydial

**Figure 2:**
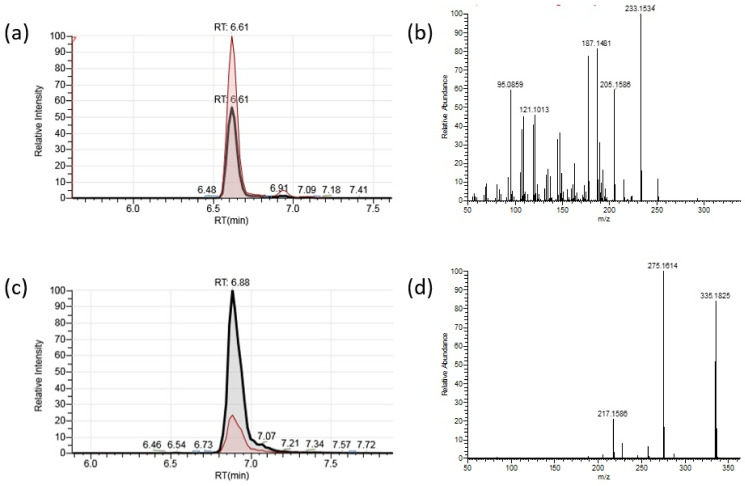
HRAM UPLC-MS/MS analysis of (**a**) botrydial [M+H]^+^ showing overlay of parallel reaction monitoring (PRM) transitions for quantification (*m*/*z* 311.1853 → 205.1586) and confirmation (*m*/*z* 311.1853 → 187.1481 ), and (**b**) the major fragmentation ions of botrydial [M+H]^+^ at 35 NCE, together with (**c**) dihydrobotrydial [M+Na]^+^ overlay of PRMs for quantification (*m*/*z* 335.1829 → 275.1614) and confirmation (*m*/*z* 335.1829 → 217.1586) and (**d**) the major fragmentation ions of dihydrobotrydial [M+Na]^+^ at 35 NCE.

HRAM UPLC-MS/MS analysis of the mixed botrydial/dihydobotrydial standard provided two peaks with molecular ions consistent with the molecular formula of the two components botrydial (**1**) (C_17_H_27_O_5_; [M+H]^+^; Calc. 311.1853 Obs. 311.1858) and dihydrobotrydial (**2**) (C_17_H_28_O_5_Na; [M+Na]^+^; Calc. 335.1829 Obs. 335.1825) (Figure 2). Dihydrobotrydial did not provide a reliable molecular ion [M+H]^+^ in ESI+ mode, and [M+Na]^+^ was selected for analysis of this metabolite. MS^2^ fragmentation ions for each metabolite were selected from predominant ions in fragmentation spectra for each component (Figure 2).

For botrydial in ESI+ mode, the major MS/MS fragmentation ions of [M+H]^+^ *m*/*z* 311.1853 (Figure 2b and Figure S1) corresponded to sequential losses: [M+H-AcOH]^+^ (C_15_H_23_O_3_ Calc 251.1647 Obs. 251.1640), [M+H-AcOH-H_2_O]^+^ (C_15_H_21_O_2_ Calc 233.1542 Obs. 233.1534), further loss of CO (C_14_H_21_O_1_ Calc 205.1592 Obs. 205.1586) and an additional loss of either H_2_O to C_14_H_19_ (Calc 187.1487 Obs. 187.1481) or a second CO to C_13_H_21_ (Calc 177.1643 Obs. 177.1637). Such losses are consistent with a protonated form of the botrydial structure (**1**). For dihydrobotrydial in ESI+ mode the major MS/MS fragmentation ions of [M+Na]^+^ *m*/*z* 335.1825 (C_17_H_28_O_5_ Calc. 335.1829) (Figure 2d) corresponded to sequential losses [M+Na-AcOH]^+^ (C_15_H_24_O_3_Na Calc 275.1623 Obs. 275.1614), further loss of NaOH and H_2_O (C_15_H_21_O_1_ Calc 217.1592 Obs. 217.1586). Such losses are consistent with the dihydrobotrydial structure (**2**), and demonstrate attachment of the Na^+^ ion within the hydroxy lactol arrangement of this compound.

Parallel reaction monitoring (PRM) transitions for botrydial (**1**) of *m*/*z* 311.1853 → 205.1586 and for dihydrobotrydial (**2**) of *m*/*z* 335.1829 → 275.1614 were selected for quantification, with secondary transitions utilized for confirmation of botrydial (**1**) (*m*/*z* 311.1853 → 187.1481 and *m*/*z* 311.1853 → 233.1534) and dihydrobotrydial (**2**) (*m*/*z* 335.1829 → 217.1586). The calibration curves based on these quantification PRM responses were linear over the range 1.02–9.92 µg/L for botrydial and 9.22–89.28 µg/L for dihydrobotrydial. Rapid separation (<7 min), good reproducibilities (average RSDs < 10%), and good recoveries (77.2% and 83.5%) were obtained in the HRAM UPLC-MS/MS analysis of both botrydial (**1**) and dihydrobotrydial (**2**) from spiked agar plates (Table 1).

### 2.3. The Effect of Different Curcumin Concentration on Curcumin-Mediated Photosensitization of B. cinerea

**Figure 3:**
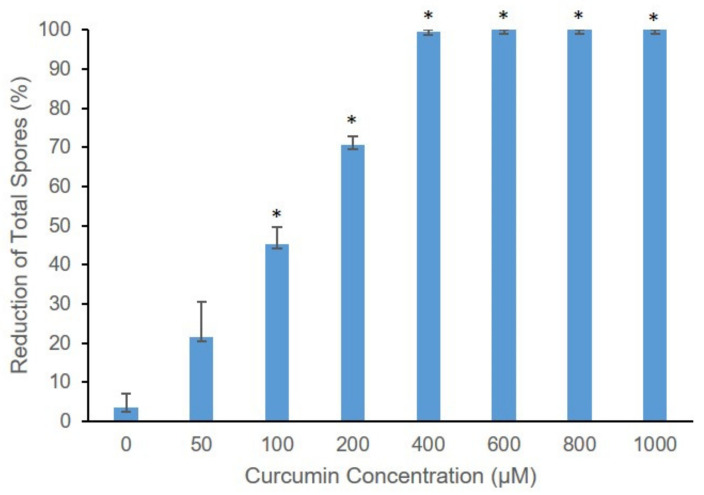
Effect of curcumin-mediated photosensitization on *B. cinerea* spore germination with different curcumin concentrations (0–1000 µM) and light dose 120 J/cm^2^ as represented by percentage reduction in total spores after incubation at 26 °C for 8 days. The significance of difference in mean colony counts (CFU) between different curcumin concentration and control (D^−^L^−^) are indicated by asterisk (*); where *p* < 0.001 tested with *t-*test.

The effect of increasing curcumin dye (D) concentrations with a photosensitization light dose (L) of 120 J/cm^2^ on *B. cinera* growth on agar plates incubated at 26 °C for 8 days was investigated. The *B. cinerea* control D^−^L^−^ treatment (D^−^ = no curcumin; L^−^ = no light) and D^−^L^+^ treatment (no curcumin, light dose 120 J/cm^2^) produced characteristic prolific greyish-brown spores, and visible differences in fungal growth was readily apparent with increasing curcumin concentrations after photosensitization treatment and incubation for 8 days (Figure S2). With increasing curcumin concentration, the growth of *B. cinerea* decreased. When treated with a curcumin concentration above 800 µM and a light does of 120 J/cm^2^, the fungus was not able to survive (Figure S2). As shown in Figure 3, a significant percentage (*p* < 0.001) reduction in the spores was also observed when the spores were treated with curcumin-mediated photosensitization. Treatment with light or curcumin alone (D^−^L^+^ or D^+^L^−^) was not effective. The curcumin-mediated photosensitization (D^+^L^+^) reduction in spore germination was directly proportionate to curcumin concentrations, with increasing concentrations of curcumin photosensitizer acting to drastically inhibit *B. cinerea* conidia germination. With curcumin concentrations above 600 µM and light dose of 120 J/cm^2^, spore germination was completely (100%) inhibited (*p* < 0.001) (Figure 3).

**Figure 4:**
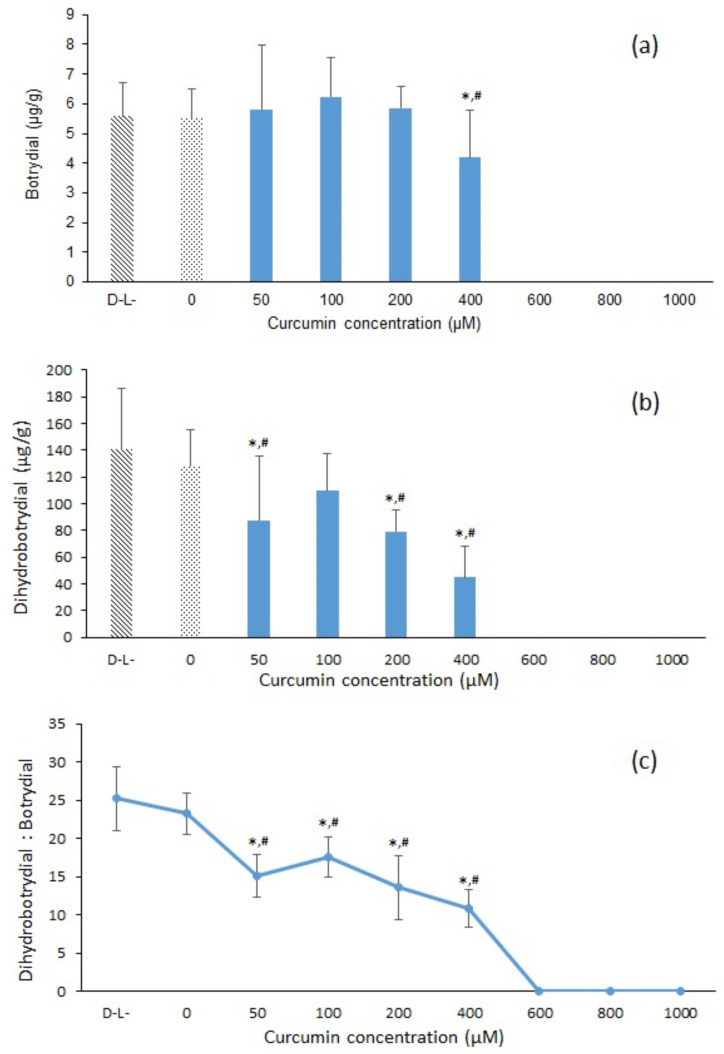
Effect of curcumin-mediated photosensitization with different curcumin concentrations (0–1000 µM) and light dose (120 J/cm^2^) on the concentration of botrydial and dihydrobotrydial in spores after incubation at 26 °C for 11 days (*n* = 6). The significances of difference in concentration of (**a**) botrydial and (**b**) dihydrobotrydial and ratio of (**c**) dihydrobotrdial/botrydial between different curcumin concentrations and light-only treatment (D^−^L^+^, curcumin = 0 µM) *p* < 0.05 (*), and compared to the control (D^−^L^−^) *p* < 0.05 (^#^), tested with *t*-test.

As shown in Figure 4a, increasing curcumin concentration from 0 to 200 µM in the photosensitization treatment resulted in no significant change in botrydial concentration after 11 days incubation at 26 °C, although there was an overall trend of decreasing dihydrobotrydial (Figure 4b). However, with a curcumin concentration of 400 µM, the fungal production of both botrydial and dihydrobotrydial was significantly reduced (*p* < 0.05), compared to control or light alone (D^−^L^−^ or D^−^L^+^). The control treatment (D^−^L^−^) contained botrydial and dihydrobotrydial at measured concentrations of 5.57 ± 1.13 µg/g and 140.50 ± 45.87 µg/g, respectively, providing a dihydrobotrdial/botrydial ratio of 25:1 (Figure 4c). The ratio of dihydrobotrdial/botrydial decreased with increasing curcumin concentration reaching a ratio of 10.3:1 with a curcumin concentration of 400 µM (Figure 4c). With curcumin concentration of 600 µM or greater at light dose of 120 J/cm^2^, no production of either botrydial or dihydrobotrydial was detected (Figure 4).

### 2.4. Effect of Curcumin-Mediated Photosensitization on the B. cinerea Spore Using Different Light Doses

**Figure 5:**
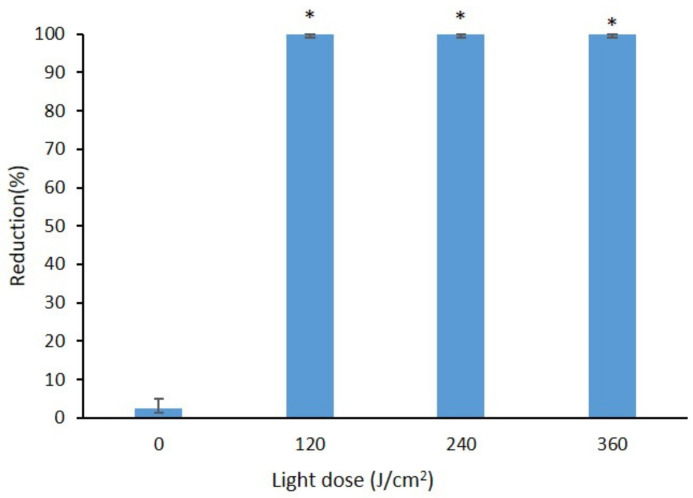
Effect of photosensitization mediated curcumin on the *B. cinerea* spore with curcumin concentration of 800 µM using different light dose (0, 120, 240, 360 J/cm^2^) as represented by percentage reduction in total spores after incubation at 26 °C for 11 days. The significance of difference in mean number of colonies between different light dose and control (D^−^L^−^) for each light dose are indicated by asterisk where (*); *p* < 0.001 tested with *t*-test.

**Figure 6:**
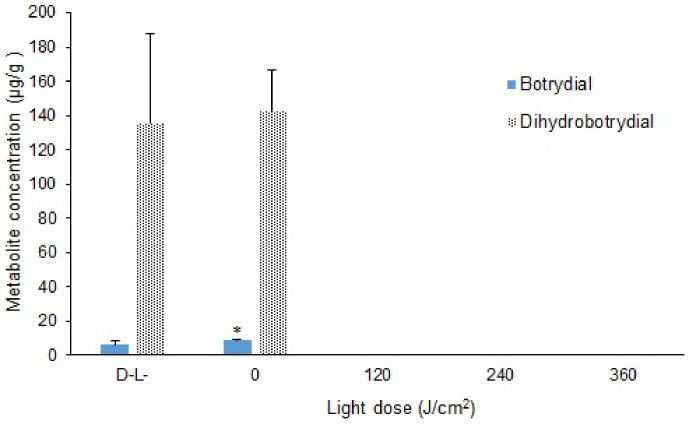
The concentration of botrydial and dihydrobotrydial in the different light dose treatments (0, 120, 240, 360 J/cm^2^) with curcumin concentration of 800 µM after incubation at 26 °C for 11 days. The significance of difference in concentration of botrydial and dihydrobotrydial between control (D^−^L^−^) and only curcumin (D^+^L^−^, light dose 0 J/cm^2^) (*n* = 9) are indicated by asterisk where (*); *p* < 0.05, tested with *t*-test. Neither botrydial nor dihydrobotrydial was detected with light doses of 120, 240 or 360 J/cm^2^.

As shown in Figure S3, when treated with a light dose of 120 J/cm^2^ and curcumin concentration of 800 µM, no spore germination was observed, which indicates that the spores were completely inactivated by this photosensitization mediated curcumin treatment. By comparison, the spores grew well after treatment with 800 µM curcumin without light (D^+^L^−^) and under control conditions (D^−^L^−^). The percentage reduction in total spores reached 100% with the curcumin-mediated light doses of 120, 240, and 360 J/cm^2^ (Figure 5). Similarly, neither botrydial nor dihydrobotrydial could be detected when light and dye (D^+^L^+^) are used at the same time with light doses of 120, 240 and 360 J/cm^2^ (Figure 6). The botrydial concentration was observed to increase slightly from 6.2 ± 2.2 to 8.5 ± 0.9 µg/g in the curcumin-only treatment (D^+^L^−^), comparing to the control (D^−^L^−^) (*p* < 0.05). However, the dihydrobotrydial concentration was not significantly different between the curcumin-only treatment (D^+^L^−^) and the control (D^−^L^−^) (Figure 6).

### 2.5. The Growth of B. cinerea over Different Incubation Time After Photosensitization

**Figure 7:**
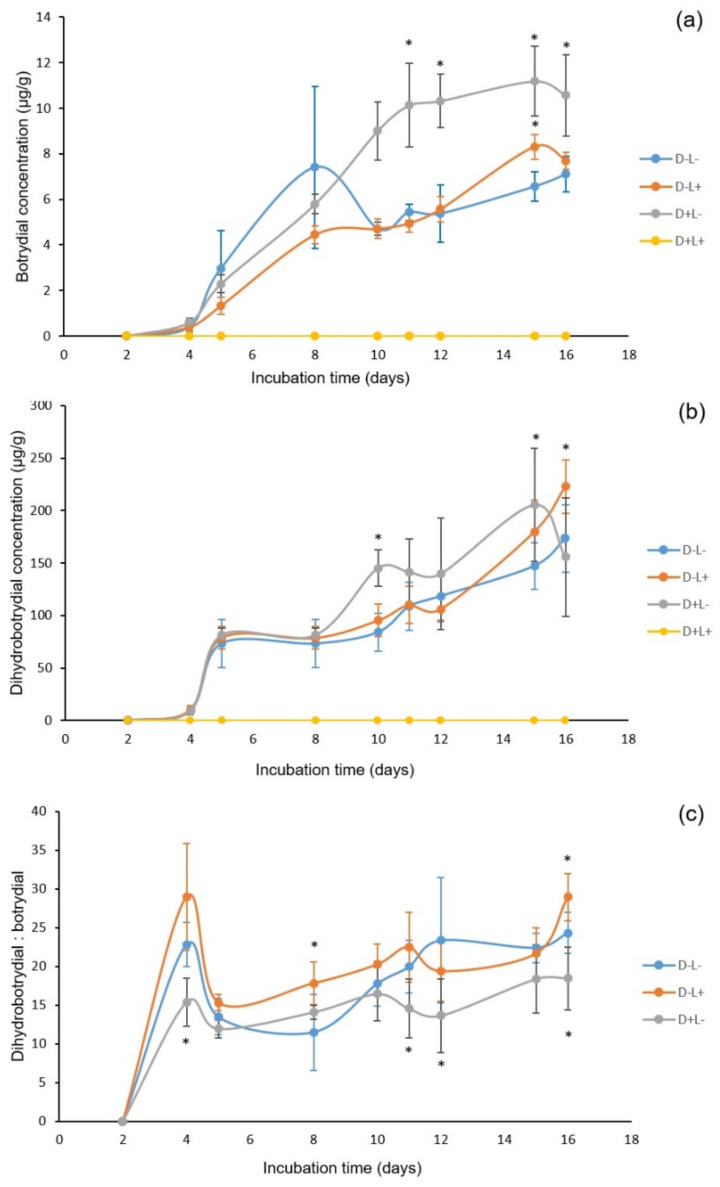
The concentration of botrydial and dihydrobotrydial after using different treatment regimes of D^+^L^+^: curcumin (800 µM) and light (120 J/cm^2^); D^−^L^−^ (control): no curcumin and no light; D^−^L^+^: no curcumin and light (120 J/cm^2^); D^+^L^−^: curcumin (800 µM) and no light followed by incubation at 26 °C for 0–16 days. The significances of difference in concentration of botrydial and dihydrobotrydial between treatment and D^−^L^−^ (control) (*n* = 6) are indicated by asterisk where (*); *p* < 0.05, tested with *t*-test. (**a**) Botrydial concentration in different treatment; (**b**) Dihydrobotrydial concentration in different treatment; (**c**) The ratio of dihydrobotrydial and botrydial concentrations in different treatment, D^+^L^+^ not included as neither metabolite detected.

In studies of effect of different treatments on *B. cinerea* with increasing incubation time (Figure S4), it was observed that in the control treatment (D^−^L^−^) mycelial growth was fast with white hyphae apparent within two days incubation. With prolonging incubation time, the three treatments (D^−^L^−^; D^−^L^+^; D^+^L^−^) display the expected regular fungi morphology. In contrast, with the curcumin treatment (D^+^L^+^), the spores did not germinate during the 16 days incubation. As shown in Figure 7, in general the light-only and dye-only treatments (D^−^L^+^) and (D^+^L^−^) demonstrated a general increase in production of both secondary metabolites reaching 8.31 ± 0.54 and 11.18 ± 1.53 µg/g of botrydial, and 179.69 ± 30.04 and 205.29 ± 53.71 µg/g of dihydrobotrydial, respectively, at 15 days. The control treatment (D^−^L^−^) produced a more variable botrydial concentration (Figure 7) with a maximum of 7.41 ± 3.56 µg/g. At all incubation times, all samples from treatment (D^+^L^+^) were below limits of detection for both botrydial and dihydrobotrydial (Figure 7), which is most likely due to the destruction of all *B. cinerea* spore by this treatment. In order to eliminate interference due to variability between plates, the ratio of dihydrobotrydial/botrydial was also calculated. The average metabolite ratio in the three treatments (D^−^L^−^; D^−^L^+^; D^+^L^−^) is 19.42 ± 3.62, 21.83 ± 3.52 and 15.34 ± 3.20, respectively.

## 3. Discussion

### 3.1. LC-MS/MS Analysis of Metabolites Botrydial and Dihydrobotrydial

High-resolution accurate mass (HRAM) spectrometers, such as the time-of-flight (TOF) and the Orbitrap spectrometer, has become more accessible and affordable to many laboratories in recent years. One of the biggest strengths of a HRAM instrument is its ability to acquire accurate mass data, which allows the user to relate the observed accurate mass to an equivalent elemental composition/s and molecular formula/s. Molecular formulas alone, however, are not sufficient to identify specific compounds. Without applicable standards, erroneous identifications can result due to the many possible compounds with the same molecular formula, and for this reason we have chosen to isolate authentic botrydial/dihydrobotrydial to use as standards in this study. These metabolites are not commercially available but can be obtained from *B. cinerea* cultures as described here.

Literature reports of UPLC-MS/MS analysis of similar authentic botrydial standards are lacking, but there are two recent literature reports on the detection of botrydial through the untargeted HRAM approach. Niedzwiecki et al. [18] studied the metabolomics from the human suction blister fluid and among their analytes observed the accurate masses of 333.1672 Da and 328.2118 Da which they attributed to [M+Na]^+^ and [M+NH_4_]^+^ molecular ions of botrydial. El Fellah et al. [19] analysed drinking water from Finland, gave a detailed account including MS^2^ fragmentations of the protonated molecular ion [M+H]^+^ C_17_H_27_O_5_ (*m*/*z*: 311.18 → 311.18454; 311.18 → 293.17410; 311.18 → 265.17929; 311.18 → 255.12225 and 311.18 → 237.11171) which they ascribed to botrydial. These authors used the co-detection of a second component identified on the basis of accurate mass (C_15_H_21_O_4,_ 265.1434) as abscisic acid, a compound also associated with *B. cinerea* fungi, to support the identification of botrydial in their water samples. Authentic botrydial and abscisic acid standards were stated to be not available and identification was based on ‘accurate mass method with UHPLC-HRMS’. Notably however according to SciFinder searches, there are 1648 different compounds known with molecular formulae C_17_H_26_O_5_. Each of these 1648 compounds would exhibit the same [M+H]^+^ 311.1853 Da (Calc for C_17_H_27_O_5_) but expected fragmentation would be different. On the basis of HRAM, the sequential losses reported by El Fellah et al. [19] can be ascribed as follows *m*/*z* 311.18 → 293.17410 loss of H_2_O; *m*/*z* 311.18 → 265.17929 loss of H_2_O and CO; *m*/*z* 311.18 → 255.12225 loss of CO and CO; and *m*/*z* 311.18 → 237.11171 loss of H_2_O and 2 × CO. Notably, no loss of AcOH was observed, which in itself is inconsistent with botrydial structure (as is the loss of 2 × CO without loss of H_2_O). The MS^2^ fragmentations of the metabolite detected by El Fellah et al. [19] is seemingly different to that of our authenticated botrydial (Figure 2 and Figure S1), and casts doubt on the water analysis results published in their report. Similarly the [M+Na]^+^ and [M+NH_4_]^+^ molecular ions reported by Niedzwiecki et al. [18] are not unique to botrydial and may or may not be actually botrydial.

Coincidentally, both the study by El Fellah et al. [19] and the current investigation were conducted on the Orbitrap mass spectrometer platform with similar elution solvent systems. We, however, find the HRAM approach not ideal for the detection of botrydial (without standard) as accurate mass fragmentation data in the literature is lacking to support the direct identification of botrydial by accurate mass alone. During our own investigations we frequently noted a second background contaminant in our samples and/or in our LCMS system which unexpectedly gives an accurate mass which is in agreement with the [M+H]^+^ molecular ion of botrydial under 5 ppm mass errors. This background compound did not co-elute with our authentic botrydial standard, nor did it exhibit the same MS fragmentation as our authenticated botrydial despite apparently having the same molecular formula accordingly to HRAM data. Consequently, without a botrydial standard we find it unreliable to identify botrydial based purely on the untargeted accurate mass data. Interestingly, dihydrobotrydial, which is a detoxification by-product of botrydial, was not reported from the two previous HRAM studies [18,19]. With the obvious gap in the untargeted HRAM detection of botrydial, we have isolated the two diagnostic metabolites, botrydial and dihydrobotrydial, from *B. cinerea* and have provided here detailed HRAM and MS^2^ accurate mass data for future comparison (Figure 2 and Figure S1). The new analysis method described here also demonstrated good reproducibility and reliability in recoveries of both botrydial and dihydrobotrydial from spiked agar plates (Table 1), enabling the use of this method to study effects of photosensitization on *B. cinerea* metabolite production.

### 3.2. Effect of Curcumin-Mediated Photosensitization on B. cinerea

This study demonstrated that the use of curcumin followed by irradiation with blue light (430 nm) promoted a significant reduction in the germination of *B. cinerea*. This result is in agreement with previous studies that have reported similar effects of curcumin and other photosensitizers with different food-borne pathogens. For example, Penha et al. [4] reported that curcumin (75 μM) and blue illumination (470 nm) had synergistic effect on inactivation of food-borne pathogens with relative order of efficacy *Aeromonas hydrophila* > *Salmonella aureus* > *Escherichia coli* > *Salmonella typhimurium.* Josewin et al. [20] similarly utilized blue light-emitting diodes (405–460 nm) with sodium chlorophyllin as photosensitizer to inactivate *Listeria monocytogenes* and *Salmonella* spp., minimizing the contamination risk of food-borne pathogens by consumption of cantaloupe. These authors achieved similar reductions with and without added photosensitizer on cantaloupe rind and speculated about the presence of an endogenous photosensitizer [20]. Our research group found that curcumin combined with visible light (420 nm) was an effective treatment to inactivate spores of *Aspergillus flavus* both in vitro and in vivo in maize kernels [5,21]. In subsequent studies, the percentage reduction in *Aspergillus niger*, *A. flavus*, *Penicillium chrysogenum* and *Zygosacharomyces bailii* remained above 50% with curcumin concentrations (100 to 400 μM) followed by illumination with a light dose of 96 J/cm^2^ [22]. In the present study, at low curcumin concentration (50 to 400 μM) and under a similar light dosage, the percentage reduction in *B. cinerea* varied from 21.5 to 99.7%, which may reflect the differing susceptibility of the fungal species studied. *Penicillium griseofulvum* by comparison demonstrated less susceptibility in previous studies [22], than that recorded here for *B. cinerea*. In all cases, the phototoxic effect seems to depend on the curcumin concentration, which is in agreement with the previous reports [23]. Fungi are usually enveloped by a thick rigid cell wall, which is made of chitin, mannoproteins and α-β glucans, and photoinactivation is therefore dependent on photosensitizer uptake through this cell wall and distribution to subcellular targets [24]. The phototoxicity of curcumin is then directed through reactive oxygen species formed by the reaction between the light-activated dye molecules and the excited states of oxygen molecules [25]. Sanita et al. [26] reported that photodynamic therapy mediated by curcumin associated with LED light significantly reduced the metabolism of the biofilm organized cells of *Candida dubliniensis*. They also reported that exposure to curcumin alone in the highest concentrations (30 and 40 μM) significantly reduced the biofilm viability. Further, while curcumin was rapidly taken up by *Candida dubliniensis* cells, curcumin was reported to require a longer time interval to penetrate into biofilm cells [26]. Araujo et al. [23] reported that curcumin has a toxic effect on cariogenic pathogens only at appreciable concentrations (5.0 g/L) upon photoactivation. Providentially, curcumin is a food additive (E100) and a naturally polyphenol extracted from *Curcuma longa* with no significant reported toxicity, well-known for its beneficial biological activities, including antimicrobial anti-proliferative, anti-inflammatory and anti-oxidant activities [27,28]. From an economic viewpoint, curcumin can be produced in high quantities at a reasonable cost, so it is an ideal photosensitizer. However, curcumin is a very hydrophobic molecule and requires some kind of formulation vehicle to enable higher curcumin load and improve interaction between dissolved curcumin and cell membranes [29]. For consumer acceptance any change in flavour and colour of the treated commodity as a result of curcumin addition would also need to be considered.

The present study also evaluated the effect of different exposure time or light dose on curcumin-mediated photosensitization of *B. cinerea* and demonstrated that only a short 10 min period of illumination was required. The D^+^L^+^ treatment achieved complete inactivation of *B. cinerea* when utilizing 800 µM curcumin and illumination time of 10 min, equivalent to a light dose of 120 J/cm^2^ (Figure 5). This result is analogous to previous studies with other microbial species. For example, Penh et al. [4] reported that with increasing the illumination time (10 to 30 min) or light does (139 to 417 J/cm^2^), curcumin-mediated photosensitization induced a significant reduction in the counts of Gram-positive and Gram-negative bacteria, such as *S. aureus*, *Pseudomonas aeruginosa* and *Aeromonas hydrophila*. Similarly, Araujo et al. [23] reported that a curcumin illumination time of 2 or 5 min was equally effective in reducing *Streptococcus mutans* and *Lactobacillus acidophilus*. It is, however, worth noting that some researchers have observed photobleaching with consequent decreases in effectiveness with increased photosensitization illumination times. For example, in evaluation of photodynamic therapy using a curcumin solution on root canals contaminated with *Enterococcus faecalis*, curcumin as sensitizer was effective with 5 min irradiation but not with 10 min irradiation, which the authors related to curcumin photobleaching [30].

This current study aimed at the identification of the most effective curcumin-mediated photosensitization treatment against *B. cinerea*. Therefore, the *B. cinerea* morphology was observed for a period of 16 days after treatment with a light does of 120 J/cm^2^ and curcumin concentration of 800 µM. In comparison to curcumin-only or light-only treatment (D^+^L^−^ and D^−^L^+^), this study demonstrated that curcumin-mediated photosensitization (D^+^L^+^) not only influences the mycelial growth of *B. cinerea*, but also causes a delay or inhibition in the germination of the fungal spore population. Curcumin has been reported to exhibit antifungal activity against *Cryptococcus neoformans*, *Phytophthora infestans*, *Rhizoctonia solani*, *Candida albicans*, and *Erysiphe graminis* [31,32]. In addition, phototherapy has been suggested as a potential therapeutic alternative to antifungal treatment for the treatment of *C. albicans* biofilm infections. Many studies have demonstrated that blue light (a wavelength of 400–500 nm) alone exhibits significantly antimicrobial effects against methicillin-sensitive *S. aureus* [33], *Acinetobacter* [34] and *C. albicans* [35]. However, in the present study, neither curcumin nor light alone was effective in eliminating *B. cinerea*, which is a similar conclusion to the previous reports with *A. flavus* [21].

The exact mechanism of the photosensitizer inducer photodynamic inactivation has not been elucidated. However, many researchers believe that the photosensitization method is based on combined action of photosensitizer, visible light, and oxygen, producing the observed cytotoxic effect [36], in which light-activated photosensitizers transfer energy to oxygen to form reactive oxygen species and other free radicals which inactivate microorganisms by damaging proteins, cell membranes, and organelles [37].

In the present study, the effects of photosensitization on *B. cinerea* phytotoxin production were also considered. Phytotoxins, including alkaloids, terpenes, polyketides, non-ribosomal peptides, or metabolites of mixed biosynthetic origin, are secreted by necrotrophic pathogens to induce cell necrosis and leakage of nutrients [38]. *B. cinerea* is one of most broad-host range fungal necrotrophs, and infects a large number of vegetables and fruit crops with subsequent economic loss both pre- and post-harvest. *B. cinerea* produces phytotoxins, ROS, as well as cell wall-degrading enzymes to induce necrosis of plant tissues. *B. cinerea* is also reported to trigger hypersensitive responses and ROS production in the host, which cause a series of programmed cell death to promote the infection process [39]. Botrydial is the primary phytotoxic sesquiterpene metabolite secreted by *B. cinerea*, and triggers ROS production, hypersensitive response, and expression of defence genes. Therefore, it is considered that botrydial may act not only as a phytotoxin, but also as an elicitor of plant defence responses [40,41]. For this reason, botrydial, as well as other *B. cinerea*-originated sesquiterpene metabolites, is regarded as an economically important fungal toxin for agriculturally and ornamental crops [11]. Although dihydrobotydial has only moderate phytotoxic activity at high concentrations [40], it is one of the major *B. cinerea* metabolites and co-occurs with botrydial during isolation [13]. The inhibition of the growth of *B. cinerea* has also been reported to be directly proportional to the botrydial concentration, with *B. cinerea* transforming botrydial to the less active phytotoxins such as dihydrobotrydial, botryenedial, and secobotrytrienediol [42]. Therefore, both botrydial and dihydrobotrydial were chosen in the present study as detection index to investigate the function of our photodynamic treatment.

Measured metabolite concentrations in the present study did not exactly mirror the effects of photodynamic degradation of *B. cinerea* spores. With increasing concentration of curcumin between 0 and 200 µM, the botrydial concentration remained relatively unchanged despite the inhibition of fungal growth (Figure 4). However, when the concentration of curcumin was raised above 200 µM, the botrydial concentration decreased and the fungi growth was also inhibited. Interestingly the production of dihydrobotrydial was more impacted at the lower curcumin concentrations. One explanation is that fungal growth may cease or be inhibited as a result of relatively higher botrydial with lesser colonies and with the increase in the concentration of curcumin. Duran-Patron et al. [42] similarly commented on the complexity of botrydial degradation pathways, with botrydial production during initial fungal growth stages regulating growth at higher concentrations, and metabolism to less inhibitory compounds, such as dihydrobotrydial, allowing growth to resume. It is noted that the botrydial concentrations measured in our agar plates (Figure 7) are less than one tenth of the maximal botrydial levels reported in the liquid culture studies of Duran-Patron et al. [42], and have not reached the levels reported by Duran-Patron et al. to regulate fungal growth. On the other hand, light-only (D^−^L^+^) or curcumin-only (D^+^L^−^) treatments may also effect the *B. cinerea* metabolite production, as seen in the varied ratio of dihydrobotrydial and botrydial produced (Figure 7). Lineiro et al. [43] found that there is a connection between gene expression of virulence factors and *B. cinerea* culture and environmental conditions, whereby the botrydial biosynthesis can be inhibited when the culture uses a sole carbon, such as cellulose and tomato cell walls. Similarly, when the spore suspension was treated with either light or curcumin in this study, this change in *B. cinerea* culture conditions could lead to a changed relationship between the metabolites botrydial and dihydrobotrydial (and consequently the ratio of these metabolites).

In the food industry, the most widely employed postharvest fruit protection methods to reduce microbial spoilage include chemicals and physical methods such as controlled atmosphere, low temperature or modified atmosphere packaging. Photosensitization as an environmentally friendly method, represents a potential novel technology with application in food and beverage industries. De Oliveira et al. [44] reported that combination of UV-A light and curcumin can significantly reduce bacterial cross-contamination of fresh produce. Tao et al. [45] reported curcumin-based photosensitization inactivate *E. coli* on the surface of apple slices and preserve the quality of apple slices. Our in vitro study has demonstrated the effectiveness of curcumin-mediated photosensitization against *B. cinerea*, a common fruit and vegetable pathogen. Further studies are required to investigate the effectiveness of this treatment against *B. cinerea* in fresh fruit such as strawberries. Promisingly, recent studies in Australian-grown strawberries demonstrated that photosensitization extended the shelf-life and did not affect the physicochemical quality of the strawberry and retained key quality attributes [9].

## 4. Conclusions

The observed reduction in *B. cinerea* spore germination was directly proportionate to curcumin concentrations, and both botrydial and dihydrobotrydial decreased with increasing curcumin concentration. In the treatment of D^+^L^+^ with an illumination time of 10 min at curcumin concentration of 800 µM, the spores were completely inhibited and these botryane secondary metabolites could not be detected even after 16 days incubation. Under the other three treatments (D^−^L^−^, D^−^L^+^ and D^+^L^−^), the spores grew normally, with both botrydial and dihydrobotrydial being produced throughout the 16 days incubation period. Curcumin-mediated photosensitization therefore represents a potentially effective method to control *B. cinerea* infection, and warrants further investigation in vivo in susceptible fruit and vegetables.

## 5. Materials and Methods

### 5.1. Fungal Materials

Five filamentous fungi (*Botrytis cinerea*, *Pestalotiopsis theae*, *Cladosporium* sp., *Penicillium raistrickii* and *Mucor rudolphii)* were isolated and identified from four commercial varieties (Fortuna, Festival, Ruby Gem and Red Rhapsody) of strawberries grown on a Queensland farm, Australia. A randomly selected sample of ten fruits was washed thrice with potable water, and drained on clean paper towels. Then, each fruit was cut into four equal pieces using a sterilized scalper, and placed on potato dextrose agar (PDA) (Thermo Fisher Scientific, Victoria, Australia) plates. The plates were incubated at 25 °C. The samples were observed daily in a laminar flow cabinet for any fungal growth, and fungi were isolated as they appear on the cut pieces using sterile needles. *B. cinerea* isolated from these strawberries was identified by colony morphology. Pure cultures were observed under a light microscope (Leica, Germany) after staining with cotton blue lactophenol [46]. Identification was confirmed by performing 18S rDNA analysis, as previously described [47]. Specifically, the fungus was cultured on Czapek Dox agar (Thermo Fisher Scientific, Victoria, Australia) at 26 °C for 15 days, and the spores were harvested by flooding the plate with 10–15 mL 0.1% Tween 80 solution. The spore solution was then filtered through sterilized double folded cheesecloth to remove the hyphal fragments. The spore solution was diluted with sterile distilled water, and concentration determined by plating 0.1 mL aliquots of ten-fold dilutions on Dichloran Rose-Bengal Chloramphenicol Agar (DRBC) (Thermo Fisher Scientific, Victoria, Australia). The spores were stored in 15% glycerine at −20 °C. For the photosensitization experiments, spore suspensions of 10^4^ CFU per mL were used, and cultured on Czapek Dox agar.

### 5.2. Isolation of Botrydial and Dihydrobotrydial Standards

Isolation of fungal metabolites was based on an adaption of the method of Lineiro et al. [43]. *B. cinerea* was grown on PDA culture for 3 days and then transferred to individual 500 mL reagent bottles (20 bottles, 3 × 1 cm plugs of agar, ca. 0.75 g per bottle), each containing about 250 mL modified Czapek–Dox medium, which were continuously shaken at 250 rpm under constant room temperature (22 °C) with 12 h light/dark cycles for 13 days. All the liquid broths were combined (ca. 4.8 L) and then extracted with ethyl acetate (3 × 1 L) (Merck, New South Wales, Australia). The combined organic extract was dried over anhydrous Na_2_SO_4_ (Merck, New South Wales, Australia) and concentrated under reduced pressure to obtain ca. 0.3 g oily residue. This residue was then subjected to silica flash column chromatography, eluting with an increasing polarity of hexane (Merck, New South Wales, Australia) and ethyl acetate mixtures from 100% hexane to 100% EtOAc. Flash column fractions (37 × 10 mL each) were collected and monitored by silica TLC under UV (254 nm) with vanillin and 2,4-dinitrophenyl-hydrazine (DNP) staining reagent applied to the air-dried TLC plates. Several fractions which were eluted with ~70% hexane were subsequently combined and evaporated to provide a solid residue (10 mg), which was confirmed by HRAM UPLC-MS/MS and ^1^H and ^13^C NMR (as described below) to contain a mixture of botrydial (**1**) and dihydrobotrydial (**2**).

### 5.3. NMR Analysis of Botrydial and Dihydrobotrydial Standards

The botrydial/dihydrobotrydial mixture (10 mg) was analysed by ^1^H and ^13^C NMR in deuterated chloroform (Cambridge Isotope Laboratories, Inc.) on a 500 MHz Bruker Avance instrument (5 mm Selective Excitation Inverse (SEI) probe) with addition of a measured aliquot of dioxane as internal standard for NMR quantification. Integration of aldehyde and dioxane ^1^H NMR resonances was used to determine the amount of botrydial present in the sample. The sample containing botrydial (0.8 mg) and dihydrobotrydial (7.2 mg) was recovered from the NMR solvent (CDCl_3_), and used without further purification as a mixed external standard in HRAM UPLC-MS/MS analysis as described below.

### 5.4. Photosensitizer and Light Source

A stock solution (2000 µM) of natural curcumin was prepared by dissolving 73.8 mg curcumin (Sigma Aldrich, St. Louis, MO, USA) in 50 mL propylene glycol (99.5%, Sigma Aldrich, St Louis, MO, USA), which was then diluted 1:1 in sterile water (50 mL). Our preliminary study showed that this solution had no inhibition effect on the spores (data not shown). The curcumin stock solution was stored in a dark cool place, and diluted as appropriate with sterile water to provide required curcumin concentrations for photosensitizer experiments. The illumination source was a 500-Watt xenon arc lamp (Polilight, PL 500, Rofin Australia Pty Ltd., Victoria, Australia) equipped with an optical fibre light with a range of 370–680 nm filters. This source was configured according to the previously described method [22] and a wavelength of 430 nm was selected. The light dose (J/cm^2^) was calculated as irradiation time (s) multiplied by the light power (w) divided by the area of irradiation (cm^2^).

### 5.5. Determination of Spore Survival under Differing Photosensitization Conditions

A wide concentration range of curcumin (50, 100, 200, 400, 600, 800, and 1000 μM) together with different levels of light doses (0, 120, 240, and 360 J/cm^2^, corresponding to an illumination time of 0, 10, 20 and 30 min, respectively) was utilized to determine an efficient photosensitization mediated curcumin treatment regime. Aliquots (1 mL) of both the fungal suspension and curcumin solution (1:1 *v*/*v*) were mixed in a Petri dish (30 mm diameter), and the light source was placed approximately 10 cm above the surface of the Petri dish. A magnetic stirrer bar was placed in the mixture (2 mL) to ensure constant stirring during illumination to expose all spores uniformly to the light energy. After illumination, aliquots (100 μL) of the treated mixture were transferred onto Czapek Dox agar (3 replicates per treatment) and incubated with 12 h light/dark cycles at 26 °C for 8 days to determine the colony forming units (CFU), with plates incubated under the same condition for a further 3 days for botrydial and dihydrobotrydial analysis. The percentage reduction in fungal spores after treatment was calculated by the number of the colony (CFU) as given below, with the results of 3 replicates averaged for each treatment.

$$\%\mathrm{Reduction}\;=\;\left({\mathrm{CFU}}_{\mathrm{control}}\;-\;{\mathrm{CFU}}_{\mathrm{test}}\right)/{\mathrm{CFU}}_{\mathrm{control}}\;\times \;100$$

The selected optimized curcumin concentration and light dose were then utilized to investigate the effect of curcumin-mediated photosensitization on *B. cinerea* spores using treatment regimes of D^+^L^+^: curcumin (800 µM) and light (120 J/cm^2^); D^−^L^−^ (control): no curcumin and no light; D^−^L^+^: no curcumin and light (120 J/cm^2^); D^+^L^−^: curcumin (800 µM) and no light followed by incubation at 26 °C for 2–16 days.

### 5.6. Determination of Toxin Production under Differing Photosensitization Conditions

*B. cinerea*-inoculated agar plates after photosensitization treatment and incubation (as described above) were also analysed by LC-MS/MS to determine botrydial and dihydrobotrydial production. A 1 cm diameter plug of agar, with an average weight of 0.25 g, was selectively removed from the respective *B. cinerea*-inoculated agar plates and extracted with acetonitrile (2.5 mL) by sonication for 10 min. The sonicated sample was then filtered (0.2 μm polypropylene membrane syringe filter) and analysed by HRAM UPLC-MS/MS with results reported as µg botrydial or dihydrobotrydial per g of agar extracted. From each agar plate, three plugs were analysed as replicates.

Spiked recovery samples for metabolite analysis were prepared by addition of aliquots of the standard botrydial/dihydrobotrydial solution to 1 cm diameter plugs of agar from un-inoculated agar plates at three different concentrations (*n* = 4), which were then extracted as above.

### 5.7. HRAM UPLC-MS/MS Conditions

UPLC separations were conducted out on an UltiMate 3000 RS UPLC system (Thermo Fisher Scientific, Bremen, Germany) equipped with an RS pump, a temperature control column compartment and an autosampler. An Agilent ZORBAX Eclipse Plus C_18_ (2.1 mm × 100 mm; 1.8 µm) column was used for the LC separations. The UPLC parameters consisted of column oven at 20 °C, an injection volume of 2.0 µL, a flowrate of 0.2 mL/min and gradient elution with mobile phase (MP) A: 0.1% formic acid in H_2_O and MP B: 0.1% formic acid in MeOH (MP B). The mobile phase gradient was 5% MP B (*t* = 0) increasing to 40% MP B at 0.8 min, 80% MP B at 1.5 min, 95% MP B at 2.2 min before reaching 100% MP B at 4 min and held there until 7 min. MP B decreased to 5% at 7.3 min and was maintained at 5% until 10 min.

The HRAM MS/MS was acquired on a Q-Exactive mass spectrometer (ThermoFisher Scientific, Bremen, Germany) with a heated electrospray ionization (HESI) probe. The MS data were collected in the parallel reaction monitoring (PRM) positive mode at 35,000 FWHM mass resolution, AGC target at 2.0e5, maximum IT at 100 ms, mass isolation window of 4.0 m/z, Normalized Collision Energy (NCE) at 35 and an inclusion list containing botrydial (C_17_H_27_O_5_, [M+H]^+^ 311.1853) and dihydrobotrydial (C_17_H_28_O_5_Na; [M+Na]^+^ 335.1829). The source settings were set as follows: spray voltage at 3500 (+), capillary temperature at 250 °C, sheath gas at 45 (arbitrary units), Aux gas flow rate at 10, Aux gas heater temperature at 400 °C, S-lens RF level at 50 and Sweep gas flow rate at 2. A 5 ppm mass accuracy window was set for the mass extraction and detection setting. The HRAM UPLC-MS/MS system was controlled by Xcalibur™ 4.1 software and the MS data were processed using TraceFinder™ 4.1 software.

External standards in the range 1.02–9.92 µg/L botrydial and 9.22–89.28 µg/L dihdrobotrydial were used for quantification with PRM transitions for botrydial (**1**) of *m*/*z* 311.1853 → 205.1586 for quantification (and *m*/*z* 311.1853 → 187.1481 and *m*/*z* 311.1853 → 233.1534 for confirmation) and for dihydrobotrydial (**2**) of *m*/*z* 335.1829 → 275.1614 for quantification (and *m*/*z* 335.1829 → 217.1586 for confirmation).

### 5.8. Statistical Analyses

All experiments were performed in triplicate. The value were expressed as mean ± SD. The analysis of statistical difference was performed using the TTEST function in Microsoft Excel 2010. Two-tails and ‘type’ for the test were choosen by the correspondence and variance of the data with FTEST. *p* < 0.05 (significant), or *p* < 0.001 (highly significant) were applied to determine any significant differences between specific means.

## Figures and Tables

**Table 1:** Recovery of botrydial (**1**) and dihydrobotrydial (**2**) from 1 cm plugs of spiked agar as measured by HRAM UPLC-MS/MS (*n* = 4 at each concentration).

Metabolite	Spiking Level µg/g	Detected Conc. (± SD), µg/g	Recovery, (± SD), %	RSD, %
**Botrydial**	0.11	0.07 (0.01)	66.3 (10.4)	15.7
	1.02	0.84 (0.04)	81.6 (4.3)	5.3
	9.92	8.31 (0.44)	83.8 (4.5)	5.4
**Average**			**77.2 (6.4)**	**8.3**
**Dihydrobotrydial**	0.10	0.08 (0.01)	81.9 (7.0)	8.5
	0.98	0.92 (0.06)	93.5 (6.1)	6.5
	9.22	6.92 (0.31)	75.1 (3.4)	4.5
**Average**			**83.5 (5.5)**	**6.6**

## Data Availability

Data available upon request.

## Supplementary Materials

The following are available online at https://www.mdpi.com/2072-6651/13/3/196/s1, Figure S1: Expansion of the botrydial MS/MS fragmentation spectra shown in Figure 2b of the manuscript (**A**), together with further expansions of the spectra sections ranging from 80 to 150 *m*/*z* (**B**) and from 150 to 260 *m*/*z* (**C**), Figure S2: Effect of curcumin-mediated photosensitization on the inactivation of *B. cinerea* spores using increasing curcumin concentrations (0–1000 µM) with a constant light dose of 120 J/cm^2^; colonies of *B. cinerea* on Czapek Dox agar medium after incubation at 26 °C for 8 days, Figure S3: Effect of photosensitization mediated curcumin on the inactivation of *B. cinerea* spore using different light dose (0,120, 240, 360 J/cm^2^) under curcumin concentration of 800 µM; colonies of *B. cinerea* on Czapek Dox agar media after incubation at 26 ^°^C, for 8 days, Figure S4: Effect of curcumin-mediated photosensitization on the inactivation of *B. cinerea* spore using different treatment regimes, after incubation at 26 °C for 2–16 days; D^−^L^−^ (control): no curcumin and no light; D^−^L^+^: no curcumin and light (120 J/cm^2^); D^+^L^−^: curcumin (800 µM) and no light; D^+^L^+^: curcumin (800 µM) and light (120 J/cm^2^).
