# Supplementary material for: The Inactivation by Curcumin-Mediated Photosensitization of Botrytis cinerea Spores Isolated from Strawberry Fruits
Source: Toxins (Basel). 2021 Mar 9;13(3):196. doi: 10.3390/toxins13030196 (PMC8002169; doi:10.3390/toxins13030196)
Supplement: Supplementary file 1 [file toxins-13-00196-s001.pdf]

Supplementary Materials: The Inactivation by Curcumin-Mediated Photosensitization of *Botrytis cinerea* Spores Isolated from Strawberry Fruits

Li Huang, Ken W. L. Yong, W. Chrishanthi Fernando, Matheus Carpinelli de Jesus, James J. De Voss, Yasmina Sultanbawa and Mary T. Fletcher

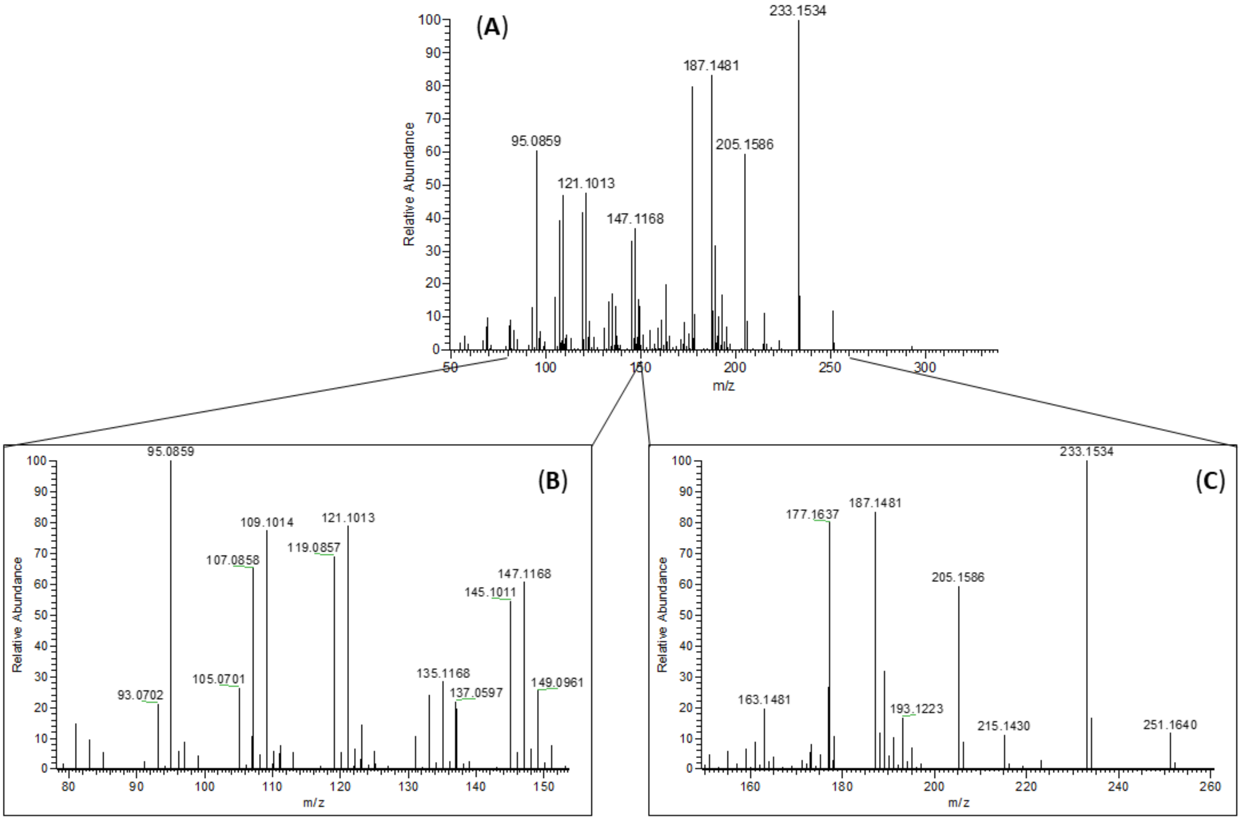

Figure S1. Expansion of the botrydial MS/MS fragmentation spectra shown in Figure 2b of the manuscript (**A**), together with further expansions of the spectra sections ranging from 80 to 150 *m*/*z* (**B**) and from 150 to 260 *m*/*z* (**C**). The major fragmentation ions *m*/*z* (relative abundance) in the full spectra (**A**) are: 95.0858 (60.7), 105.0701 (15.7), 107.0858 (40.0), 109.1014 (46.6), 119.0857 (42.8), 121.1013 (48.4), 133.1012 (14.4), 135.1168 (17.1), 137.0597 (13.0), 145.1011 (32.4), 147.1168 (36.3), 149.0961 (15.5), 163.1481 (20.1), 177.1637 (79.4), 187.1481 (82.4), 189.1637 (31.6), 193.1223 (17.8), 205.1586 (61.4), 233.1534 (100.0), 234.1567 (16.2). The molecular ion *m*/*z* 311.1853 is not detected in this spectrum.

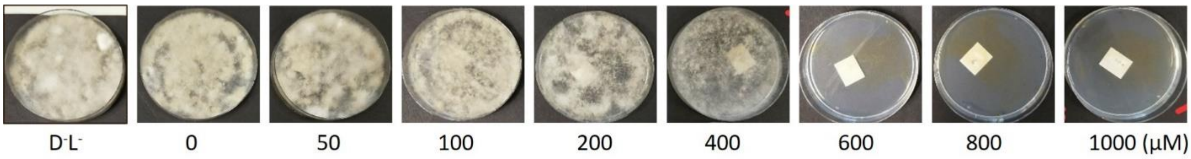

Figure S2. Effect of curcumin-mediated photosensitization on the inactivation of *B. cinerea* spores using increasing curcumin concentrations (0–1000 μM) with a constant light dose of 120 J/cm^2; colonies of *B. cinerea* on Czapek Dox agar medium after incubation at 26 °C for 8 days.

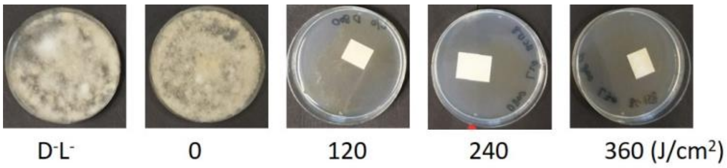

Figure S3. Effect of photosensitization mediated curcumin on the inactivation of *B. cinerea* spore using different light dose (0, 120, 240, 360 J/cm^2) under curcumin concentration of 800 μM; colonies of *B. cinerea* on Czapek Dox agar media after incubation at 26 °C, for 8 days.

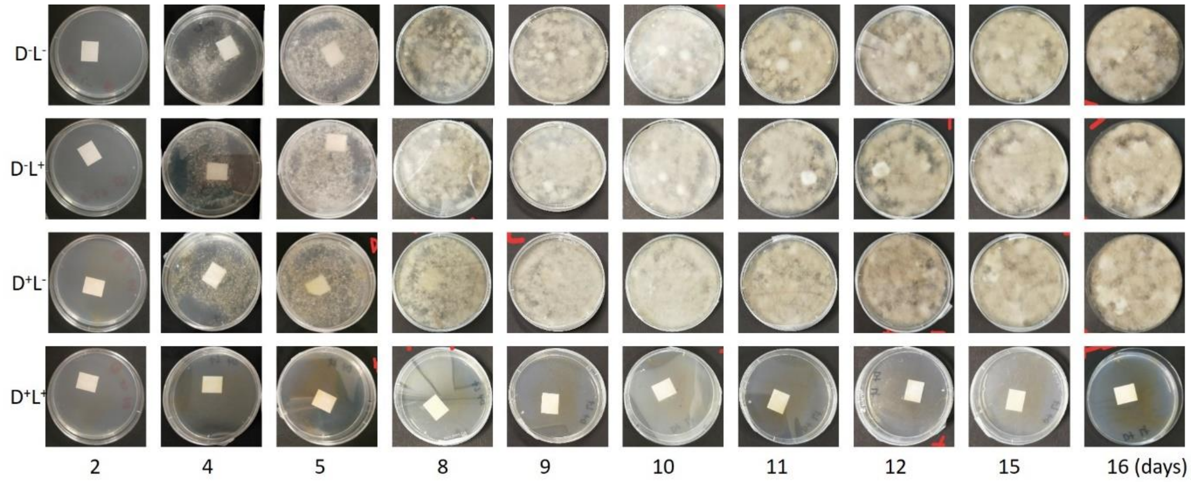

Figure S4. Effect of curcumin-mediated photosensitization on the inactivation of *B. cinerea* spore using different treatment regimes, after incubation at 26 °C for 2–16 days; D^-L^- (control): no curcumin and no light; D^-L^+: no curcumin and light (120 J/cm^2); D^+L^-: curcumin (800 μM) and no light; D^+L^+: curcumin (800 μM) and light (120 J/cm^2).
